# Supplementary figures and images for: Finding novel relationships with integrated gene-gene association network analysis of Synechocystis sp. PCC 6803 using species-independent text-mining
Source: PeerJ. 2018 May 23;6:e4806. doi: 10.7717/peerj.4806 (PMC5970561; doi:10.7717/peerj.4806)

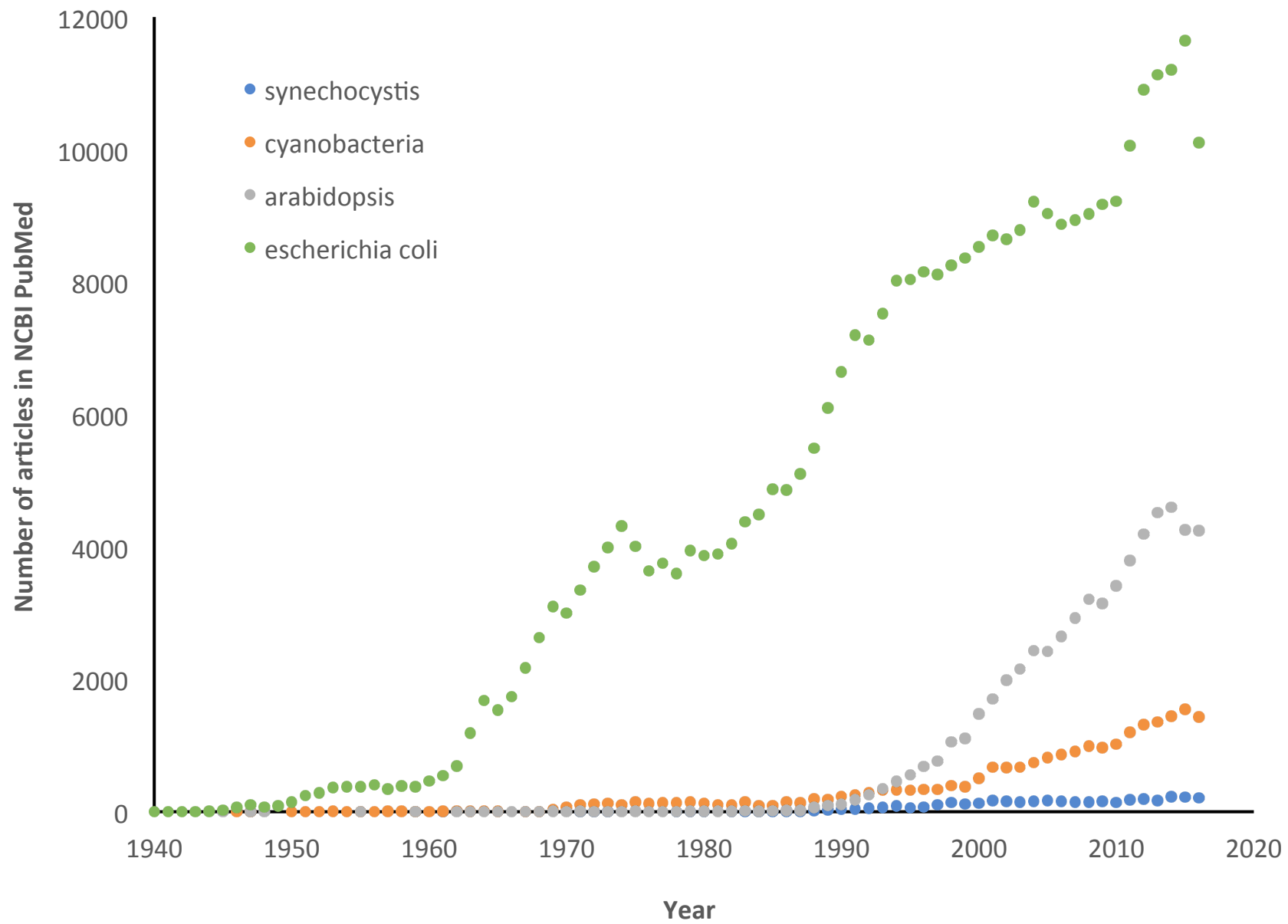

Supplement: Figure S1 — The search terms ‘Synechocystis’ (representing Synechocystis sp. PCC 6803), ‘Cyanobacteria’, ‘Arabidopsis” (representing the model plant Arabidopsis thaliana) and ‘Escherichia coli’ were entered into PubMed (http://www.ncbi.nlm.nih.gov/pubmed) July 2017. The numbers shown in the figure were obtained from this website by selecting “Results by year”. [file peerj-06-4806-s001.pdf]
